# Strip steel surface defect detection based on lightweight YOLOv5

**DOI:** 10.3389/fnbot.2023.1263739

**Published:** 2023-10-04

**Authors:** Yongping Zhang, Sijie Shen, Sen Xu

**Affiliations:** School of Information Engineering, Yancheng Institute of Technology, Yancheng, China

**Keywords:** defect detection, target detection, GSConv, SimAM, loss function

## Abstract

Deep learning-based methods for detecting surface defects on strip steel have advanced detection capabilities, but there are still problems of target loss, false alarms, large computation, and imbalance between detection accuracy and detection speed. In order to achieve a good balance between detection accuracy and speed, a lightweight YOLOv5 strip steel surface defect detection algorithm based on YOLOv5s is proposed. Firstly, we introduce the efficient lightweight convolutional layer called GSConv. The Slim Neck, designed based on GSConv, replaces the original algorithm's neck, reducing the number of network parameters and improving detection speed. Secondly, we incorporate SimAM, a non-parametric attention mechanism, into the improved neck to enhance detection accuracy. Finally, we utilize the SIoU function as the regression prediction loss instead of the original CIoU to address the issue of slow convergence and improve efficiency. According to experimental findings, the YOLOv5-GSS algorithm outperforms the YOLOv5 method by 2.9% on the NEU-DET dataset and achieves an average accuracy (mAP) of 83.8% with a detection speed (FPS) of 100 Hz, which is 3.8 Hz quicker than the YOLOv5 algorithm. The proposed model outperforms existing approaches and is more useful, demonstrating the efficacy of the optimization strategy.

## 1. Introduction

Strip steel is a crucial component in domestic construction. Due to the influence of the production process and external environmental factors, strip steel inevitably develops defects such as scratches, cracks, and pitting during processing (Zhu et al., 2023). These faulty strip steel products are subsequently sold and used in the market, posing serious dangers to worker safety and building site security. Therefore, it is crucial to research fast and accurate methods for detecting surface defects in strip steel (Yeung and Lam, 2022).

Traditional defect detection methods rely on manual visual inspection, which is inefficient, costly, poses safety hazards in the inspection environment, and lacks reliability (Damacharla et al., 2021). In particular situations, approaches for defect detection based on machine vision can produce satisfactory results. However, when applied to the complex industrial scenes, various environmental and lighting effects can lead to a decline in the accuracy of the detection results. Research on strip steel surface defect detection techniques based on deep learning has progressively entered the mainstream in recent years due to the rapid advancement of deep learning technology (Kou et al., 2021).

Deep learning-based target detection algorithms can be classified into two categories: the first category consists of two-stage detection algorithms, such as R-CNN (Girshick et al., 2014) and Faster R-CNN (Ren et al., 2016). He et al. (2017) implemented a multiscale fusion end-to-end network for inspecting strip surfaces and created the NET-DET dataset for strip surface defect detection. Zhao et al. (2021) improved the Faster R-CNN network by replacing part of the conventional convolutional network with a variable convolutional network, resulting in a 0.128 improvement in detection accuracy. The second category comprises single-stage detection algorithms, including the YOLO (Redmon et al., 2016) and SSD (Kong et al., 2017) algorithms. Kou et al. (2021) enhanced the YOLOv3 model by incorporating anchorless techniques for detecting targets with significant scale variations, and the dense structure of the algorithm led to substantial improvements in handling high-resolution data. Li et al. (2020) improved YOLOv3 by employing a weighted K-means clustering algorithm and introducing a large-scale detection layer, achieving an 80% increase in detection accuracy. Mi et al. (2023) proposed a novel data augmentation method based on YOLOv4 to enhance the robustness and accuracy of the algorithm's detection capabilities. Liu et al. (2023) designed the MSC-DNet model, which excels in pinpointing defects and significantly contributes to the accurate detection of medium and large-scale defects. Fu et al. (2023) introduced a multi-scale pooled convolutional neural network-based approach for steel surface defect detection and classification. This method utilized pre-trained models and transfer learning techniques, which allowed training with limited samples and improved the generalization ability of the model. Chen et al. (2023) proposed a fast detection network for strip surface defects called DCAM-Net, based on deformable convolution and attention mechanism. Experimental results on the NEU-DET dataset demonstrated an mAP of 82.6% for this algorithm, coupled with a high detection speed of 100.2 FPS, effectively enhancing the efficiency of strip steel surface defect detection.

Despite having increased detection capabilities, the aforementioned techniques nevertheless run into problems such target loss, false alarms, excessive computational demands, and an unbalanced balance between detection precision and speed. To strike a better balance between detection accuracy and speed, this paper introduces YOLOv5-GSS, a lightweight strip steel surface defect detection algorithm based on YOLOv5. The main contributions of this paper are highlighted as follows:

- To reduce the number of parameters and the computational burden of the model, we incorporate GSConv (Li et al., 2022) into the neck network for lightweight enhancement. By utilizing GSConv's parallel computing capacity, this use enhances the model's computational effectiveness and speeds up its inference process. The model can learn channel associations thanks to GSConv, which also makes the model more expressive and boosts detection precision.
- To enhance detection accuracy without compromising the number of model parameters and detection speed, we integrate SimAM (Yang et al., 2021), a non-parametric attention mechanism, into the improved neck. SimAM dynamically assigns weights to the feature map, enabling the model to prioritize important features and reduce unnecessary feature computations. This method improves detection speed and accuracy by concentrating on pertinent data while minimizing computing overhead.
- In YOLOv5, the CIoU metric normalizes the distance between the predicted bounding box and the ground truth bounding box, but this normalization can introduce training instability in certain scenarios. In this paper, we replace CIoU with SIoU (Gevorgyan, 2022) because it doesn't need to be normalized. Improved training stability and increased model training effectiveness are both provided by SIoU. By using SIoU instead of CIoU, we mitigate the potential training issues caused by normalization and achieve more stable and efficient training of the model.

In summary, YOLOv5-GSS leverages GSConv, SimAM, and SIoU to achieve efficient, accurate, and stable strip steel surface defect detection while striking a balance between precision and speed.

The remaining sections of this paper are outlined as follows. Section 2 provides a comprehensive review of related work in the field. Section 3 details the architecture of our proposed method. In Section 4, we present comparative experiments and analysis to demonstrate the outstanding performance of our model. Finally, Section 5 concludes the paper and provides insights into future research directions.

## 2. Related theories

### 2.1. YOLOv5 algorithm

The YOLO model, as a prominent single-stage detection algorithm, is known for its fast operation and low memory consumption. YOLOv5 builds upon the strengths of the original YOLO model while incorporating state-of-the-art computer vision techniques to enhance detection accuracy and training speed.

The YOLOv5 network is composed of four main components: Input, Backbone, Neck, and Head. The Backbone, which serves as the feature extraction network, consists of three modules: the CBS (basic convolutional unit) module, the C3 residual network module, and the SPPF (spatial pyramidal pooling fusion) module. The CBS module performs convolutional operations, batch normalization, and activation operations. It plays a crucial role in extracting useful features from the input data. The C3 module is responsible for feature extraction and incorporates residual connectivity, allowing the network to learn complex features by combining information from different layers. This enables the network to capture and represent intricate patterns and details. The SPPF module outputs larger and more accurate feature representations. It utilizes spatial pyramidal pooling to capture both fine-grained and coarse-grained features from the input feature map. This helps improve the accuracy of the target detection task by incorporating multi-scale contextual information. Overall, the Backbone in YOLOv5 combines the capabilities of the CBS, C3, and SPPF modules to extract meaningful features from the input data, enabling accurate and efficient target detection.

The Neck component of YOLOv5 employs an FPN+PAN structure. The Feature Pyramid Network (FPN) structure, introduced by Lin et al. (2017), effectively captures and conveys semantic information, leading to improved detection performance. It achieves this by integrating feature maps from different network layers, enabling the model to handle objects of various scales. The Path Aggregation Network (PAN), proposed by Liu et al. (2018), complements the FPN by introducing a bottom-up structure. It facilitates the flow of strong localization features from lower layers to higher layers, enhancing the localization signal and further improving the accuracy of object detection. The combination of FPN and PAN in the Neck component allows the YOLOv5 model to leverage rich and diverse feature information from multiple scales, enabling more precise and comprehensive object detection. The head component, often referred to as the detection head, is a crucial element of the YOLOv5 algorithm. It performs multi-scale target detection on the feature maps extracted by the Backbone and Neck components. The Head utilizes these feature maps to generate detection results, including bounding box coordinates, class probabilities, and confidence scores. In summary, the Neck component, with its FPN+PAN structure, enhances the feature fusion process, while the Head component performs multi-scale target detection based on the extracted feature maps, ultimately providing accurate detection results in the YOLOv5 algorithm.

### 2.2. Lightweight convolution GSConv

Convolutional Neural Networks (CNNs) have the advantage of being lightweight, making them suitable for running and deploying models efficiently, especially in resource-constrained scenarios. Several common lightweight methods and techniques used in CNNs include:

- A pruning algorithm is employed to remove unimportant connections within the network, thereby reducing the number of model parameters.
- Designing lightweight network structures, such as MobileNetV3 (Howard et al., 2019), ShuffleNetV2 (Ma et al., 2018), EfficientNet (Tan and Le, 2019), etc., is another approach to reduce the computation and number of parameters in the network. These structures often utilize techniques like Depthwise Separable Convolution (DSC) and channel rearrangement. By isolating the channel information from the input image during the computation phase, DSC is helpful in lowering the number of parameters. The feature extraction and fusion capabilities of DSC may be compromised by this separation, though. As a result, the detection accuracy of the model can be significantly compromised.
- Distillation techniques are employed to train small neural networks, such as YOLOv4-tiny (Bochkovskiy et al., 2020), YOLOX-tiny (Ge et al., 2021), YOLOv7-tiny (Wang et al., 2022), etc., with the goal of achieving similar performance to the original model. By utilizing distillation, knowledge from a larger, more complex model (the teacher model) is transferred to a smaller, more lightweight model (the student model). The student model is trained to mimic the behavior and predictions of the teacher model, allowing it to achieve comparable performance while having fewer parameters and reduced computational requirements. Distillation techniques enable the development of compact models that can be deployed efficiently in resource-constrained scenarios without sacrificing performance.

**Figure 1:**
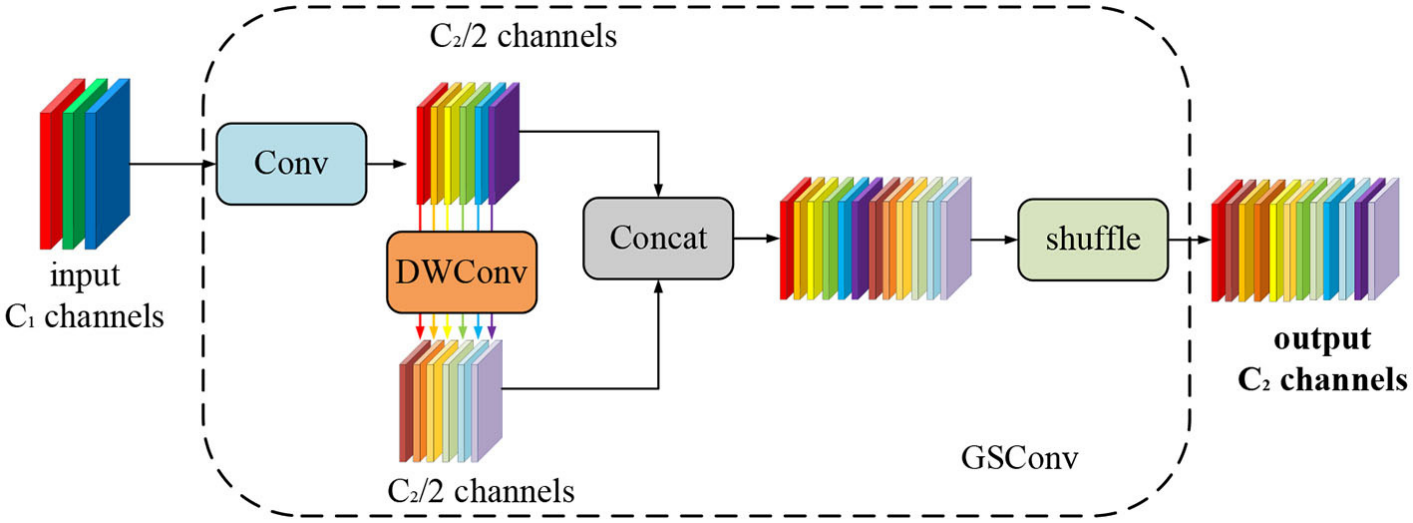
The GSConv generation process.

In contrast to the accuracy sacrifice observed in the aforementioned lightweight approaches, this paper introduces the efficient lightweight convolutional GSConv. This convolutional technique not only reduces the number of parameters and increases detection speed but also ensures improved detection accuracy. Combining traditional convolution with depth-separable convolution, GSConv applies a shuffling uniform mixing approach. It combines the strengths of both types of convolutions to enhance the expressive power of the network while reducing computational complexity. Figure 1 shows the generation process of GSConv and how it effectively and efficiently combines traditional and depth-separable convolutions.

In GSConv, the shuffle strategy is employed to permeate the information generated by conventional convolution into each part of the Depthwise Separable Convolution (DSC). This ensures that the feature information is evenly exchanged across different channels within the DSC output, without requiring additional complex measures. GSConv successfully makes up for the considerable drop in detection accuracy that could happen when DSC is used directly by including the shuffling approach. This approach ensures that the feature information from conventional convolution is mixed thoroughly with the DSC output, enhancing the model's ability to capture meaningful features and maintaining detection accuracy.

### 2.3. SimSM attention mechanism

Attention mechanisms have been shown to be effective for feature optimization in computer vision tasks by focusing limited computational resources on more important targets (Lindsay, 2020). One popular attention mechanism is the Squeeze-and-Excitation Network (SENet) (Hu et al., 2018), which employs a one-dimensional channel-based attention module with low computational complexity and high target optimization efficiency. SENet has been commonly applied in various target detection scenarios. However, its dimensionality reduction and dimensionality increase operations can result in the loss of potentially valuable feature information. To address this drawback, the proposed Efficient Channel Attention (ECA) mechanism (Wang et al., 2020) compensates for the information loss by effectively achieving cross-channel interaction. It enhances the ability of the model to capture relevant information across channels. The Coordinated Attention (CA) mechanism (Hou et al., 2021) introduces coordinates into the attention mechanism. It calculates an attention score for each location in the feature map based on the coordinates and uses these scores to weigh the features at each location. This allows the network to focus on the most relevant regions of the input image, leveraging spatial information for better attention-based feature optimization. Furthermore, researchers have proposed other attention mechanisms such as GAMAttention (Liu et al., 2021a), a global attention mechanism that spans the spatial and channel dimensions, and NAMAttention (Liu et al., 2021b), an attention mechanism based on normalization. These mechanisms offer additional ways to enhance feature optimization by considering global relationships and leveraging normalization techniques, respectively.

**Figure 2:**
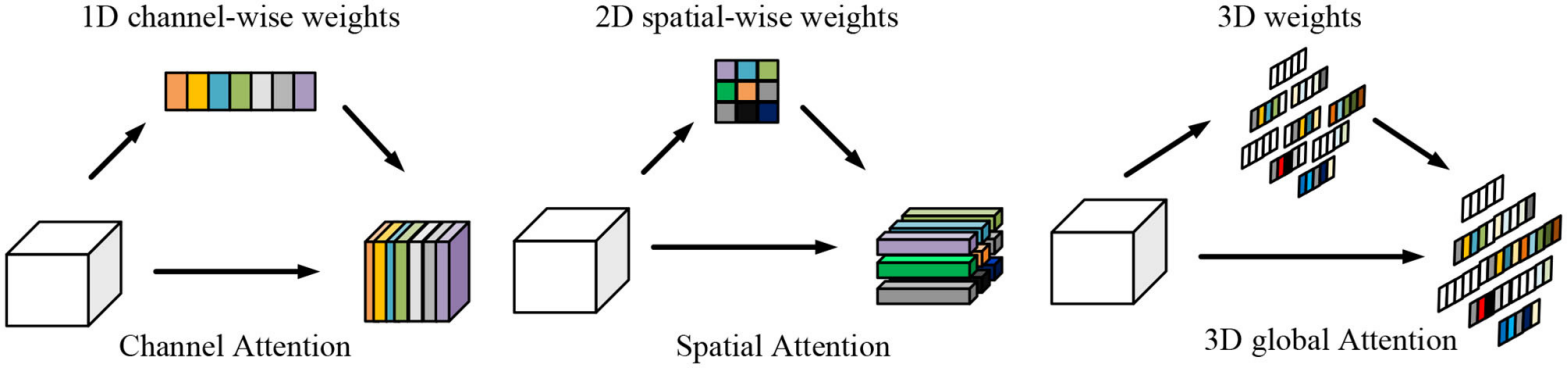
Comparison of attentional mechanisms in different dimensions.

Compared to channel attention modules and spatial attention modules, which demand additional training layers with numerous additional parameters, the SimAM attention mechanism is a straightforward, parameter-free plug-and-play module that has advantages. SimAM applies full three-dimensional weights to the attention mechanism, enhancing its effectiveness. Figure 2 illustrates the comparison of SimAM with other attention mechanisms.

The SimAM attention mechanism provides the benefit of extrapolating 3D attention weights from the feature map without introducing additional parameters into the original network. It accomplishes this by establishing an energy function based on neuroscience principles, which is employed to deduce the 3D weights from the current neuron. Subsequently, this module refines these neurons sequentially while preserving the original network structure. Equation (1) represents the energy function defined by SimAM:


(1) $$\begin{array}{l} {e_{t}}^{*}=\frac{4\left({\hat{\sigma }}^{2}+\text{λ}\right)}{{\left(t-\hat{\mu }\right)}^{2}+2{\hat{\sigma }}^{2}+2\text{λ}} \end{array}$$

In the provided equation, $\hat{\mu }$ represents the mean parameter associated with a specific location, and ${\hat{\sigma }}^{2}$ represents the variance parameter of that location, $\mu_{t}=\frac{1}{M-1}\sum_{i=1}^{M-1}x_{i}$ represents the mean of the neurons, excluding the target neuron *t*, within the input feature's single channel. Similarly, $\sigma_{t}^{2}=\frac{1}{M-1}{\left(x_{i}-\mu_{t}\right)}^{2}$ denotes the variance of the neurons, excluding the target neuron *t*, within the same channel. Here, *M* stands for the number of energy functions assigned to each channel, with *x*_*i*_ representing the target neuron and the other neurons within the single channel of the input feature. This equation demonstrates that a smaller value of the energy function $e_{t}^{*}$ indicates a higher variance between the target neuron *t* and the surrounding neurons.

### 2.4. Regression loss function

The loss function for the target detection task consists of two components: a classification loss function and a regression loss function. The regression loss function plays a significant role in improving model accuracy by facilitating better localization. To address issues such as slow convergence, low detection accuracy, and unstable model training caused by coordinate regression loss, the IoU Loss (Jiang et al., 2018) was introduced in the ACM2016 paper. It is defined as the negative logarithm of the intersection over union ratio between the predicted bounding box and the ground truth bounding box. However, IoU-based metrics have their limitations, and subsequent advancements have been made to improve their effectiveness. The following order of advancements has been proposed: Generalized IoU (GIoU) (Rezatofighi et al., 2019), Distance-IoU (DIoU) (Zheng et al., 2020), Complete-IoU (CIoU), Enriched-IoU (EIoU) (Zhang et al., 2022), and Alpha-IoU (αIoU) (He et al., 2021). These loss functions incorporate variations of bounding box regression metrics. However, they do not consider the direction of mismatch between the desired true box and the predicted box, which can result in slow and inefficient convergence. The introduction of SIoU (Scale-Invariant IoU) effectively addresses this problem by considering the direction of mismatch between the desired true box and the predicted box. SIoU has proven to be beneficial in achieving more efficient and stable convergence during training.

SIoU is a novel loss function that redefines the metric of penalty by incorporating vector pinch angles prior to expected regression. SIoU consists of four cost functions: Angel cost, Distance cost, Shape cost, and IoU cost. The overall SIoU can be expressed as the sum of the Distance cost, Shape cost, and IoU loss. Therefore,


(2) $$\begin{array}{l} total\;SIoU\;=\;Distance\;cost\;\left(angle\;+\;distance\right)+\\ \;Shape\;cost\;+\;IoU\;loss \end{array}$$

The expression for Angel cost is:


(3) $$\begin{array}{l} \text{Λ}=1-2^{*}sin^{2}\left(arcsin\left(x\right)-\frac{\pi }{4}\right) \end{array}$$

**Figure 3:**
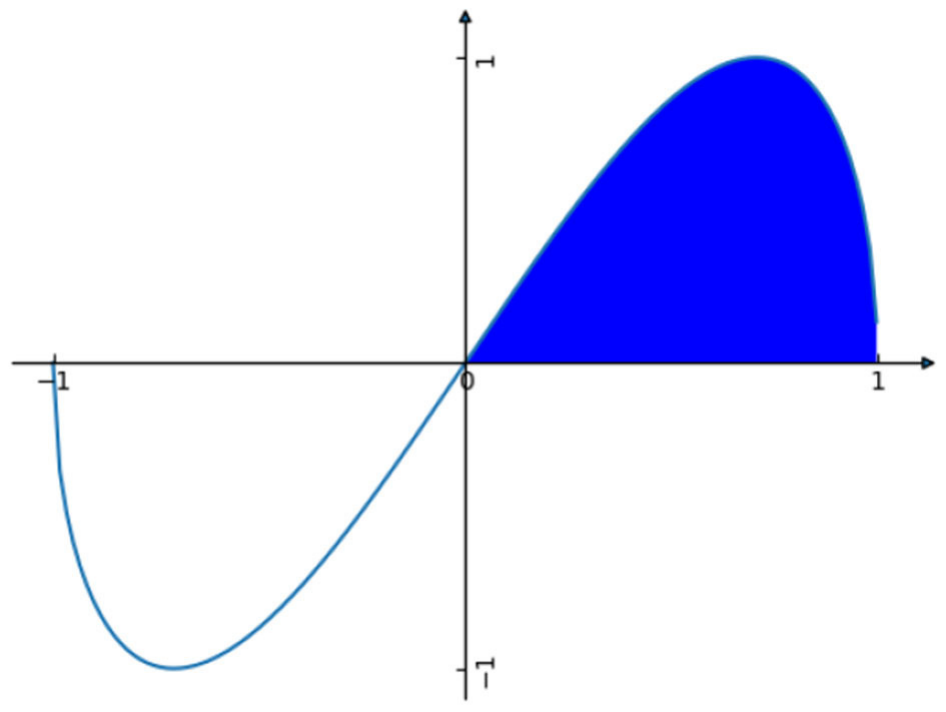
Graph of Angel cost.

The curve is depicted in Figure 3.

The Distance cost is defined by the following equation:


(4) $$\begin{array}{l} \text{Δ}=\underset{t=x,y}{\sum }\left(1-e^{-\gamma pt}\right) \end{array}$$

In the formula, we have the following definitions: $p_{x}={\left(\frac{b_{cx}^{gt}-b_{cx}}{c_{w}}\right)}^{2}$, $p_{y}={\left(\frac{b_{cy}^{gt}-b_{cy}}{c_{h}}\right)}^{2}$, γ = 2 − Λ. It can be observed that α determines the magnitude of the contribution of the Distance cost, while γ assigns a time-preferred distance value that increases with the angle.

The Shape cost is defined by the following equation:


(5) $$\begin{array}{l} \text{Ω}=\underset{t=w,h}{\sum }{\left(1-e^{-\omega t}\right)}^{\theta } \end{array}$$

In the formula, *w* and *h* indicate the width and height of the detection frame, and θ controls the degree of attention to shape loss.

The final defining equation for SIoU is as follows:


(6) $$\begin{array}{l} L_{box}=1-IoU+\frac{\text{Δ}+\text{Ω}}{2} \end{array}$$

## 3. Lightweight YOLOv5 algorithm design

### 3.1. Network structure of the YOLOv5-GSS

**Figure 4:**
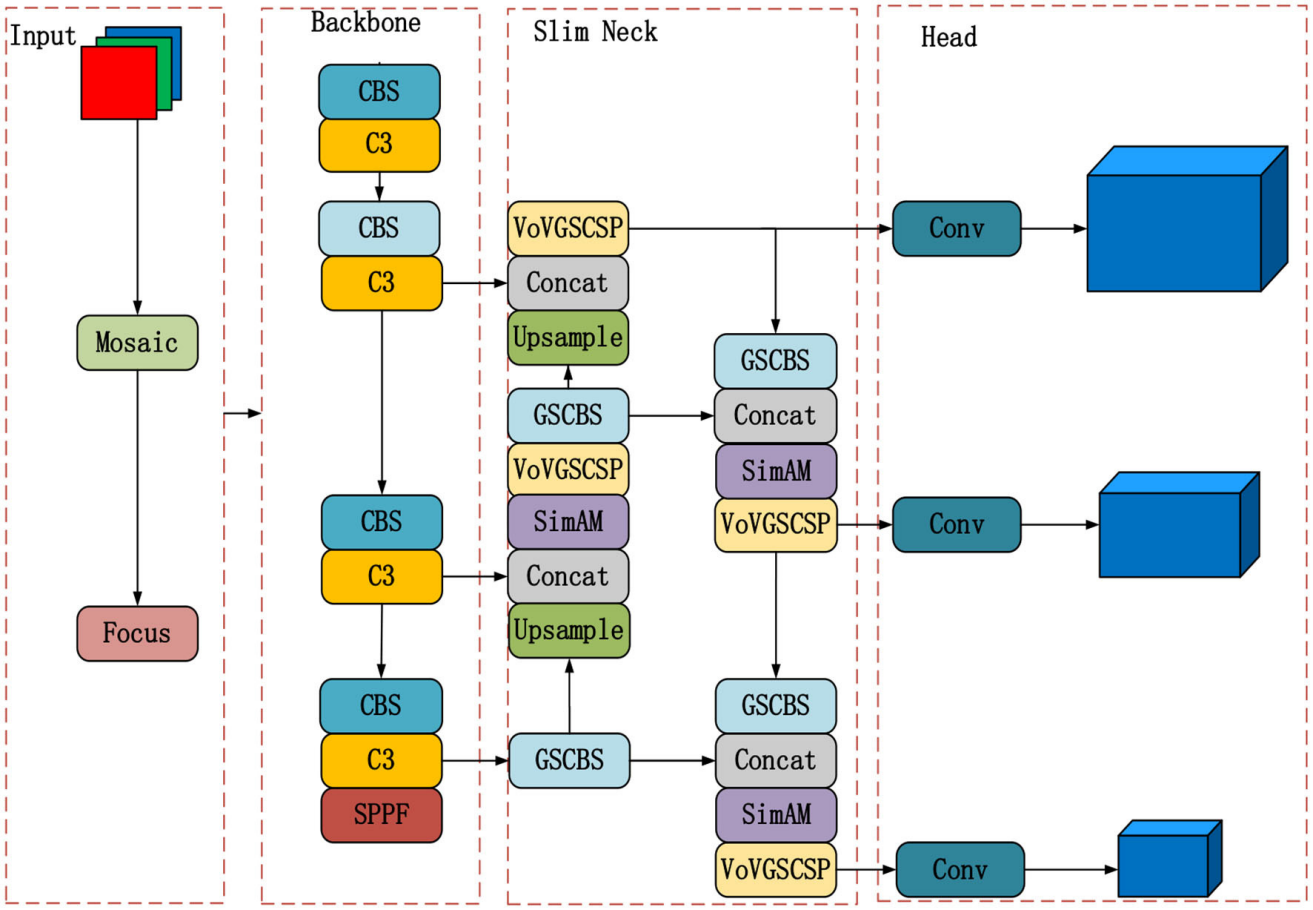
Diagram of the YOLOv5-GSS framework.

The advantage of YOLOv5 is that it successfully strikes a balance between the quantity of network parameters and detection precision. It still has issues with low detection accuracy and slow detection speed, though. This study offers the YOLOv5-GSS lightweight algorithm to overcome these problems. It optimizes YOLOv5 by incorporating lightweight convolution, attention module, and regression loss function. The overall architecture of the YOLOv5-GSS algorithm comprises four main parts, as depicted in Figure 4.

Unlike YOLOv5, YOLOv5-GSS utilizes the lightweight feature fusion network, Slim Neck, and incorporates an attention mechanism. Furthermore, the regression loss function is replaced with SIoU. Overall, YOLOv5-GSS distinguishes itself from YOLOv5 by optimizing the model architecture with these enhancements. These improvements collectively reduce the network's parameter count while significantly enhancing detection speed and efficiency, making YOLOv5-GSS a superior choice for strip steel surface defect detection.

### 3.2. Slim neck construction

To ensure efficient and easy deployment of the algorithm for real-time industrial inspection of strip surface defects, YOLOv5-GSS incorporates the use of GSConv to redesign the neck, referred to as the Slim Neck structure in this paper. The Slim Neck structure, as illustrated in Figure 4, is designed to reduce computational requirements and the number of parameters in the algorithm. This optimization results in increased detection speed without compromising detection accuracy. By implementing the Slim Neck structure and leveraging the benefits of GSConv, YOLOv5-GSS aims to strike a balance between accuracy and speed, making it suitable for real-time strip surface defect detection in industrial settings.

**Figure 5:**
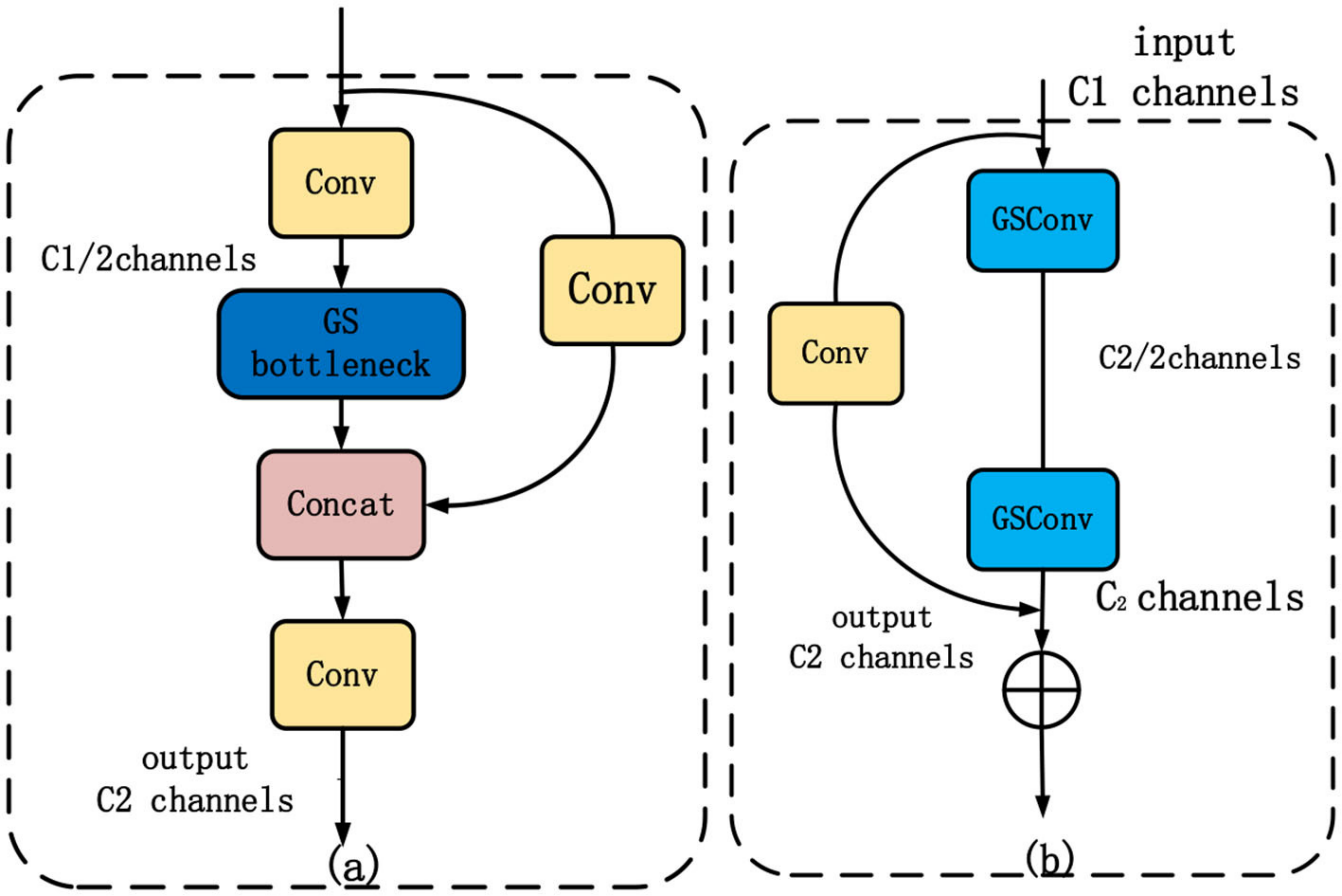
Gsbottleneck and VoV-GSCSP structure. **(a)** Depicts the architecture of VoV-GSCSP, while **(b)** represents the fundamental module GSbottleneck that constitutes VoV-GSCSP.

The base structure block of the Slim Neck, known as VoV-GSCSP, is illustrated in Figure 5a. It is a lightweight 3D convolutional structure that incorporates the GSConv and CSP structures. The GSCSP, represented as the GSbottleneck in Figure 5b, serves as the core component of the VoV-GSCSP. It consists of multiple CSP connections, a 3 × 3 convolutional layer, and the GSConv. The most significant distinction between GSCSP and the residual network module in YOLOv5 is the utilization of the GSConv layer instead of the traditional 3 × 3 convolutional layer. The GSConv layer divides the convolutional kernel into several groups and performs convolutional operations on each group.

The specific steps involved in the VoV-GSCSP block are as follows:

- First, feature extraction of the input feature map is performed using a 3 × 3 convolutional layer.
- Second, the feature map is partitioned into groups, with each group containing a specific number of channels.
- Third, spatial channel grouping convolution is applied to each group, reducing the number of channels.
- Fourth, the results from each group are concatenated to obtain a feature map that is connected across the stages.
- Fifth, feature fusion and dimensionality reduction are performed on the feature map using a 1 × 1 GSConv layer.
- Sixth, the feature map is added to the input feature map through a residual connection.

The advantages of the VoV-GSCSP structure include:

- First, the feature map is partitioned into groups, and independent spatial channel grouping convolution operations are performed on each group. This approach effectively reduces computation and the number of parameters.
- Second, the adoption of cross-stage partial concatenation helps preserve the richness and diversity of features, leading to improved accuracy.

YOLOv5-GSS incorporates GSConv in the neck to address feature mapping with reduced redundant repetitive information and without compression. This is achieved by utilizing grouped convolution, where the input feature mapping is divided into multiple smaller groups, and each group performs the convolution operation independently. As a result, the convolution operation avoids redundant computation, as each group only processes its own inputs without considering information from other groups. This approach effectively reduces the computational complexity and inference time of the network model while maintaining model accuracy. However, it's important to note that GSConv is not used throughout the entire network in YOLOv5-GSS. Utilizing GSConv at all stages of the model would increase the number of layers in the network, potentially leading to higher training errors and significantly extending the network training time. The specific validation of this approach is presented in the experimental Section 4.4.1 to ensure clarity and readability for reviewers.

### 3.3. Introduction of the SimAM attention mechanism

To enhance the detection accuracy, the parameter-free SimAM is incorporated into the Slim Neck module of YOLOv5-GSS (refer to the Slim Neck module in Figure 4). It not only computes the 3D weights between features but also accelerates the calculation and fusion of weights. The steps for weighted calculation using the SimAM attention mechanism in the Slim Neck section of YOLOv5-GSS are as follows:

Step 1: A set of 1 × 1 convolutional layers is employed to upsample or downsample the feature maps from various layers of the backbone network to a consistent resolution. The resulting feature maps are denoted as *F* = {*F*_1_, *F*_2_, ..., *F*_*N*_}, where $F_{i}\in {\mathbb{R}}^{\left(C\times H\times W\right)}$ represents the feature map of layer *i*.

Step 2: For each feature map *F*_*i*_ and *F*_*j*_, three tensors are calculated: Qi (query), Kj (key), and Vj (value).

Step 3: The attention score *S*_*i, j*_ between the *i* and *j* feature maps is calculated as follows:


(7) $$\begin{array}{l} S_{i,j}=Q_{i}\times K_{j} \end{array}$$

Step 4: The attention weight *a*_*i*_ of the *i* feature map is calculated using the following formula:


(8) $$\begin{array}{l} a_{i}=softmax\left(\underset{j}{\sum }S_{i,j}\right) \end{array}$$

where softmax is a function.

Step 5: The weighted feature map for the *ith* feature map is calculated by:


(9) $$\begin{array}{l} F_{i}^{\prime }=\underset{j}{\sum }\left(a_{j}\times V_{j}\right) \end{array}$$

where *a*_*j*_ is the attention weight of the *jth* feature map.

Step 6: The weighted feature map $F_{i}^{\prime }$ is combined with the original feature map to obtain the output features *F*_*s*_.

In summary, the incorporation of the SimAM attention mechanism module enhances the feature fusion capability of the network without increasing the number of network parameters. This ensures efficient computational power of the network while effectively improving the detection accuracy of the algorithm.

### 3.4. Regression loss function

The purpose of the loss function is to minimize the discrepancy between the predictions of the model and the true target position. In YOLOv5-GSS, the loss function comprises three components: regression loss, confidence loss, and classification loss. After replacing the regression loss function with SIoU, the complete loss function of the algorithm is:


(10) $$\begin{array}{l} L_{object,Loss}=L_{box,Loss}+L_{conf,Loss}+L_{class,Loss} \end{array}$$

where: *L*_*conf, Loss*_ represents the confidence loss, and *L*_*class, Loss*_ represents the classification loss.

Both confidence loss and classification loss are calculated using a binary cross-entropy loss function. The expression for the cross-entropy loss function is shown below:


(11) $$\begin{array}{l} C=-\frac{1}{n}\underset{x}{\sum }\left[ylna+\left(1-y\right)\ln\left(1-a\right)\right] \end{array}$$

where *x* represents the sample, *y* represents the actual label (*y*=*0 or 1*), *a* represents the predicted output, expressed as the probability that the predicted sample belongs to category *1*, and *n* represents the total number of samples. The term *ylna* indicates that the model predicts a loss in category *1* when the sample actually belongs to category *1*, while *(1-y)ln(1-a)* indicates that the model predicts a loss in category *0* when the sample actually belongs to category *0*. The sum of the loss functions *C* is the sum of the losses of all samples. The cross-entropy loss function measures the difference between the predicted probability distribution and the true probability of a class. By minimizing the classification loss, the ability of network to accurately classify objects in an image can be improved, thereby enhancing overall detection performance.

In summary, the loss function algorithm for YOLOv5-GSS can be summarized as Algorithm 1:

**Algorithm 1:**
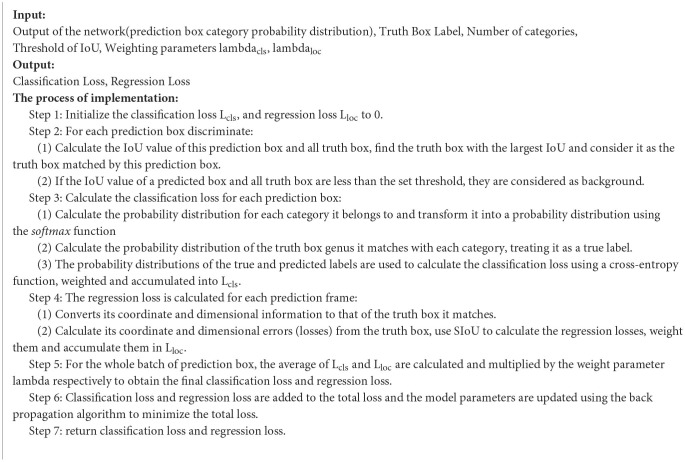
Loss function for YOLOv5-GSS

## 4. Analysis of experimental results

Four versions of the YOLOv5 are available in different sizes: YOLOv5s (smallest), YOLOv5m (medium), YOLOv5l (large), and YOLOv5x (extra-large). In this paper, the base model chosen for improvement and comparison is YOLOv5s, which is the smallest variant. The experiments conducted in this study are described below.

### 4.1. Experimental environment

The hardware setup for the experiments in this paper includes an Intel Core i5 12490F CPU with 16GB of RAM and an NVIDIA GeForce RTX 3060 GPU with 12GB of video memory. The software environment utilized is PyTorch, a deep learning framework for Windows, with Python version 3.8 and PyTorch version 1.7.1. The experimental settings and parameter configurations are showen in Table 1. The provided table outlines the parameter settings used for the experimental conditions. A batch size of 16 was employed, and training was conducted over 300 epochs. The momentum factor was set at 0.937, while the initial learning rate was initialized to 0.001. Threshold values for Intersection over Union (IoU) were chosen at 0.5, and weight decay was set to 0.0005. These parameter choices collectively define the training configuration utilized for the experiments.

**Table 1:** Experimental parameters.

**Condition parameters**	**Parameter settings**
Batch size	16
Epoch	300
Momentum factor	0.937
Initial learning rate	0.001
Threshold values for IoU	0.5
Weight decay	0.0005

### 4.2. Experimental data set

The NEU-DET (He et al., 2020) dataset, developed by Professor Song Kechen's team at Northeastern University, was selected for this paper. The dataset contains 1,800 grayscale images of hot-rolled strip steel, capturing six common surface defects: rolling swarf (Rs), plaque (Pa), cracking (Cr), pockmarking (Ps), inclusions (In), and scratches (Sc). Each defect type consists of 300 samples, resulting in a total of six classes. The images have a raw resolution of 200 × 200 pixels. It is worth noting that within each class, there can be significant variations in appearance, and there may be similarities between different classes, which poses challenges for detection tasks. To facilitate the experiments, the dataset provides annotations indicating the class and location of each defect in the images. Prior to conducting the experiments, the dataset was divided into a training set and a validation set, with a ratio of 9:1.

### 4.3. Evaluation indicators

To visually and comprehensively evaluate the performance of the improved algorithm and compare it with other detection methods, various evaluation metrics were employed, including Average Precision (*AP*) for single-class accuracy, mean Average Precision (*mAP*) for multi-class accuracy, precision (*P*), recall (*R*), Frames Per Second (*FPS*) for detection speed, Parameters (*Params*), and Giga-Floating Point Operations (*GFLOPs*) to assess computational space and time complexity. Precision (*P*) is defined as the ratio of true positive predictions to the total number of predicted positives, while recall (*R*) is the ratio of true positive predictions to the total number of actual positives. The expressions for precision and recall are as follows:


(12) $$\begin{array}{l} P=\frac{TP}{TP+FP} \end{array}$$

(13) $$\begin{array}{l} R=\frac{TP}{TP+FN} \end{array}$$

Where *TP* denotes the number of true positive predictions, *FP* denotes the number of false positive predictions, and *FN* denotes the number of false negative predictions. The Precision-Recall (*P-R*) curve is plotted using the values of precision and recall, and the area under the curve represents the Average Precision (*AP*), as shown in Equation (13):


(14) $$\begin{array}{l} AP=\int_{0}^{1}p\left(r\right)dr \end{array}$$

Where *p* and *r* denote the precision and recall rates, respectively. The mean Average Precision (*mAP*) is obtained by dividing the sum of the Average Precisions (*AP*) for each category by the number of categories *C*, as shown in Equation (14):


(15) $$\begin{array}{l} mAP=\frac{\sum_{i=1}^{C}AP_{i}}{C} \end{array}$$

### 4.4. Comparison of experimental results

#### 4.4.1. Ablation experiments

To verify the superiority of YOLOv5-GSS over YOLOv5, ablation experiments were conducted on the experimental dataset using the controlled variable method. The performance of YOLOv5-GSS with different modules was analyzed, and the validation results for each module are presented in Table 2.

- The YOLOv5 with Silm Neck block is called YOLOV5-G.
- The YOLOv5 with Silm Neck block and SimAM block is called YOLOV5-GS.
- The YOLOv5 with Silm Neck block, SimAM block and SIoU is called YOLOV5-GSS.

**Table 2:** Results of ablation experiment.

**Improve**	**mAP/%**	**Params**	**GFLOPs**	**FPS**
YOLOv5	80.9	7026307	15.8	96.2
YOLOv5-G	81.5	5849187	12.7	104
YOLOv5-GS	82.7	5849187	12.7	103
YOLOv5-GSS	83.8	5849187	12.7	100
YOLOv5+GSConv (all)+SimAM+SIoU	81.2	5085907	8.8	92.6

Table 2 demonstrates that the addition of each module resulted in improved mAP compared to the original algorithm. Firstly, replacing normal convolution with GSConv and using the Slim Neck structure designed on top of GSConv improved the neck of the network model. This modification reduced the computational effort (GFLOPs) from 15.8 to 12.7, increased the detection speed (FPS) by 7.8, and improved mAP by 0.6%. These results validate that GSConv, as a lightweight convolution, contributed to the improvement in detection speed. Secondly, the addition of the SimAM attention mechanism before the residual module in the Slim Neck structure further improved mAP by 0.8% without increasing the model parameters. This confirms the effectiveness of incorporating the attention mechanism. Lastly, using SIoU as the regression loss function without introducing additional parameters increased the detection accuracy (mAP) by 1.1%.

After the overall improvement, both the detection accuracy and speed of the algorithm have significantly improved. The analysis reveals that Slim Neck plays a crucial role in achieving these improvements. Slim Neck effectively lowers computation and parameter count by lowering the amount of feature map channels, which increases model operation speed. Furthermore, the reduction in feature map channels enhances the model's ability to learn target relationships and features, thereby improving its expressiveness and ultimately boosting detection accuracy. By enabling the model to concentrate more on the properties unique to small targets—which often exhibit fewer features—Slim Neck specifically aids in improving the detection of small targets. Additionally, Slim Neck facilitates feature reuse by reducing the number of channels between the feature extractor and the detection network. By reusing features, the model becomes more expressive and has better detecting abilities. In contrast, the original YOLOv5 architecture had separate feature extraction and detection networks, which limited feature reuse.

The SimAM attention mechanism plays a vital role in enhancing the model's ability to understand the similarities and differences between different objects, leading to improved detection accuracy. This is accomplished by applying weights to various feature maps using self-attention, accentuating features that are important for the detection task and attenuating irrelevant features. This weighting operation enhances the expressiveness of the model without introducing additional computational complexity. Additionally, the SimAM attention mechanism incorporates channel reduction techniques to decrease the dimensionality of the feature map. Model compression is made possible by this reduction in dimensionality, which also lowers computing costs and storage needs. This improves runtime efficiency. Consequently, the SimAM attention mechanism contributes to increased detection speed without sacrificing computational efficiency.

Surface defects on steel strips can come in various shapes and sizes. Some defects may be small, while others could be larger. SIOU takes into account the scale of the objects, which means it provides a fair evaluation of detection performance regardless of the object's size. This scale invariance is crucial for accurately assessing the quality of detections across the entire range of defect sizes. This attribute is particularly beneficial for detecting surface defects on steel strips, where objects can exhibit diverse scales and challenging localization conditions. As a result, using SIOU as the localization loss can lead to an observed improvement in mAP and overall detection accuracy. In summary, the combined technical improvements, including GSConv, SimAM, and SIoU, effectively enhance the detection speed and accuracy of the algorithm. These improvements are achieved by reducing computational effort, increasing the expressiveness of the model, and improving the performance of the loss function.

The validation results in Table 2 also demonstrate that GSConv is most effective when applied to the neck structure of the network. While maintaining all other factors constant, replacing every convolution method in the network with GSConv results in a considerable decrease in detection time without considerably increasing detection accuracy. The reason behind this observation is that while GSConv reduces the computation by decomposing convolution operations, it also introduces additional group convolution operations. This implies that more multiplication and addition operations are required in each convolutional layer, which can potentially increase the overall computation. Additionally, processing each channel using group convolution may produce more intricate memory access patterns. Regular convolution, on the other hand, processes each channel separately. Additionally, using GSConv in the backbone network may negatively affect memory bandwidth and caching effects, slowing down computation. These findings validate the effectiveness of the specific improvements proposed in this paper.

**Figure 6:**
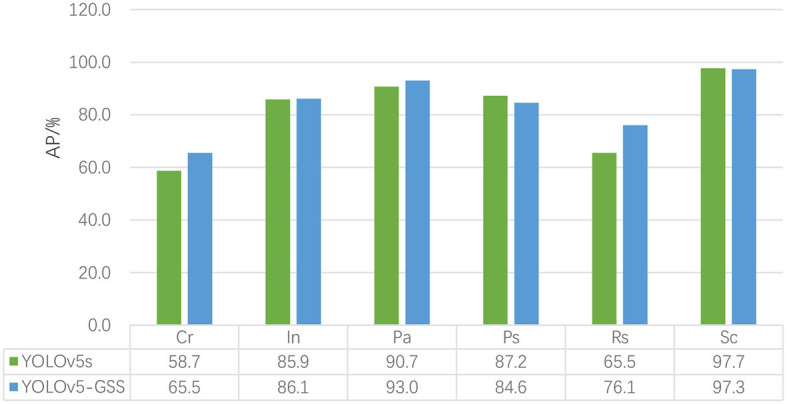
Comparison of YOLOv5 and YOLOv5-GSS. The figure includes the detection accuracy data for each defect class before and after model improvement, as well as a more visually informative bar chart for comparison.

Figure 6 presents a comparison between the YOLOv5-GSS and YOLOv5 algorithms for each category. The figure clearly illustrates the comparison of detection results across different categories. It can be observed that the accuracy has significantly improved for categories such as Cr and Rs. Although a modest decline in accuracy is seen for two other categories, there is a substantial improvement in In and Pa. This decline does not, however, prevent the overall rise in mAP.

**Figure 7:**
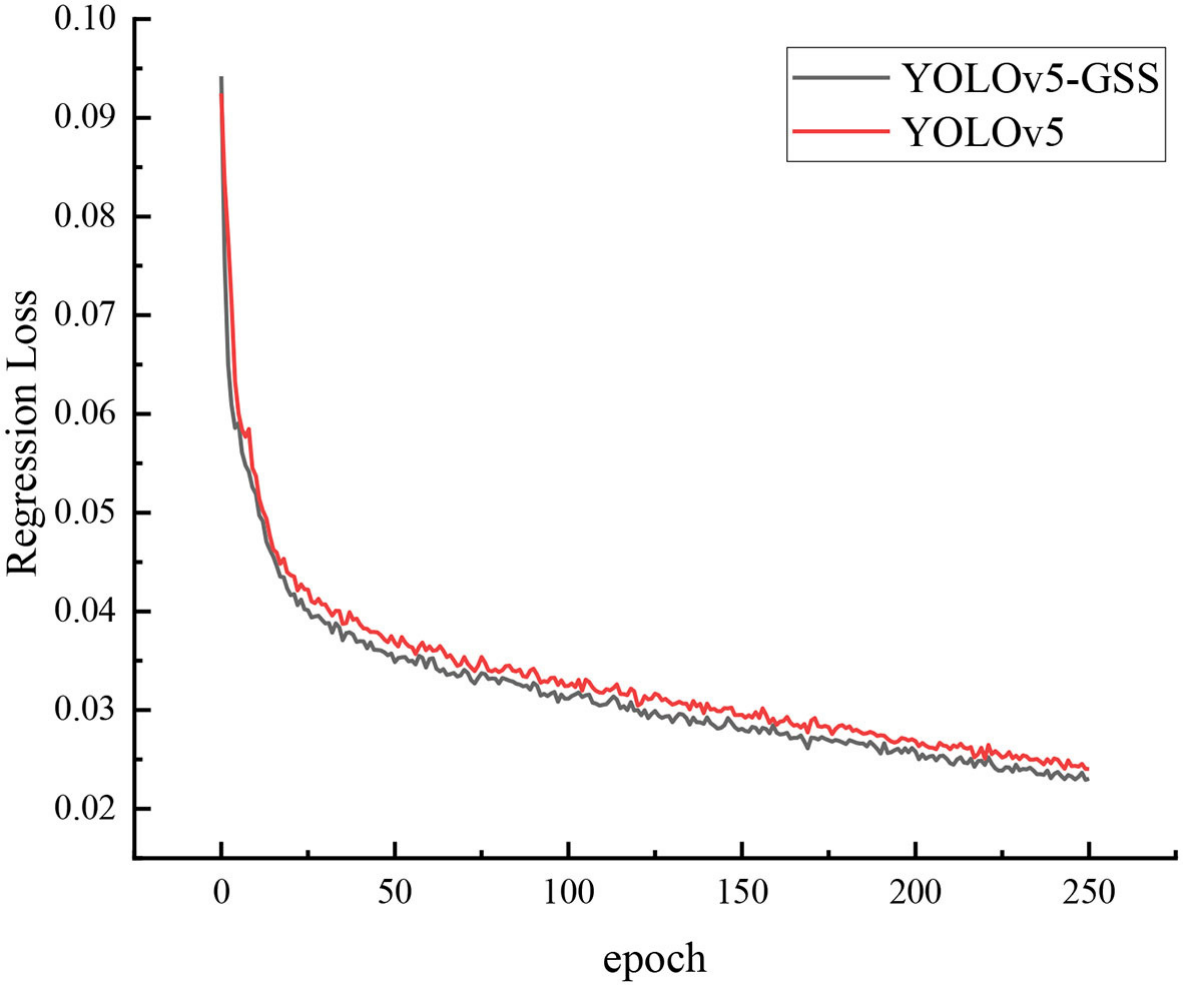
Loss curve for YOLOv5 and YOLOv5-GSS. The black curve represents the loss curve of YOLOv5-GSS, while the red curve represents the loss curve of YOLOv5. The x-axis corresponds to epochs, and the y-axis represents the value of the loss function.

Figure 7 depict the regression loss of YOLOv5 and YOLOv5-GSS. The regression loss curve is commonly employed to monitor the performance of object detection models during training. It signifies the disparity between the accuracy of the model's predicted object bounding box positions and the actual annotations. A lower regression loss curve indicates more accurate bounding box predictions by the model. Throughout the training process, the model continually adjusts its parameters using optimization algorithms to minimize the regression loss. The loss curve gradually descends as training progresses, eventually reaching convergence or a stable state. In cases of insufficient training, the loss curve may exhibit unstable fluctuations or a decelerated descent rate. In the graph, the x-axis denotes the number of training iterations or epochs, while the y-axis represents the regression loss values. Observing the regression loss curve aids in gauging the model's training progress and identifying issues like overfitting or underfitting. Since the network tends to converge prematurely before reaching 300 epochs during training, we have opted to plot the loss function graph for the first 250 epochs. From the graph, it is evident that the regression loss curve of YOLOv5-GSS descends more rapidly, indicating the clear advantage of utilizing SIOU as the regression loss function.

**Figure 8:**
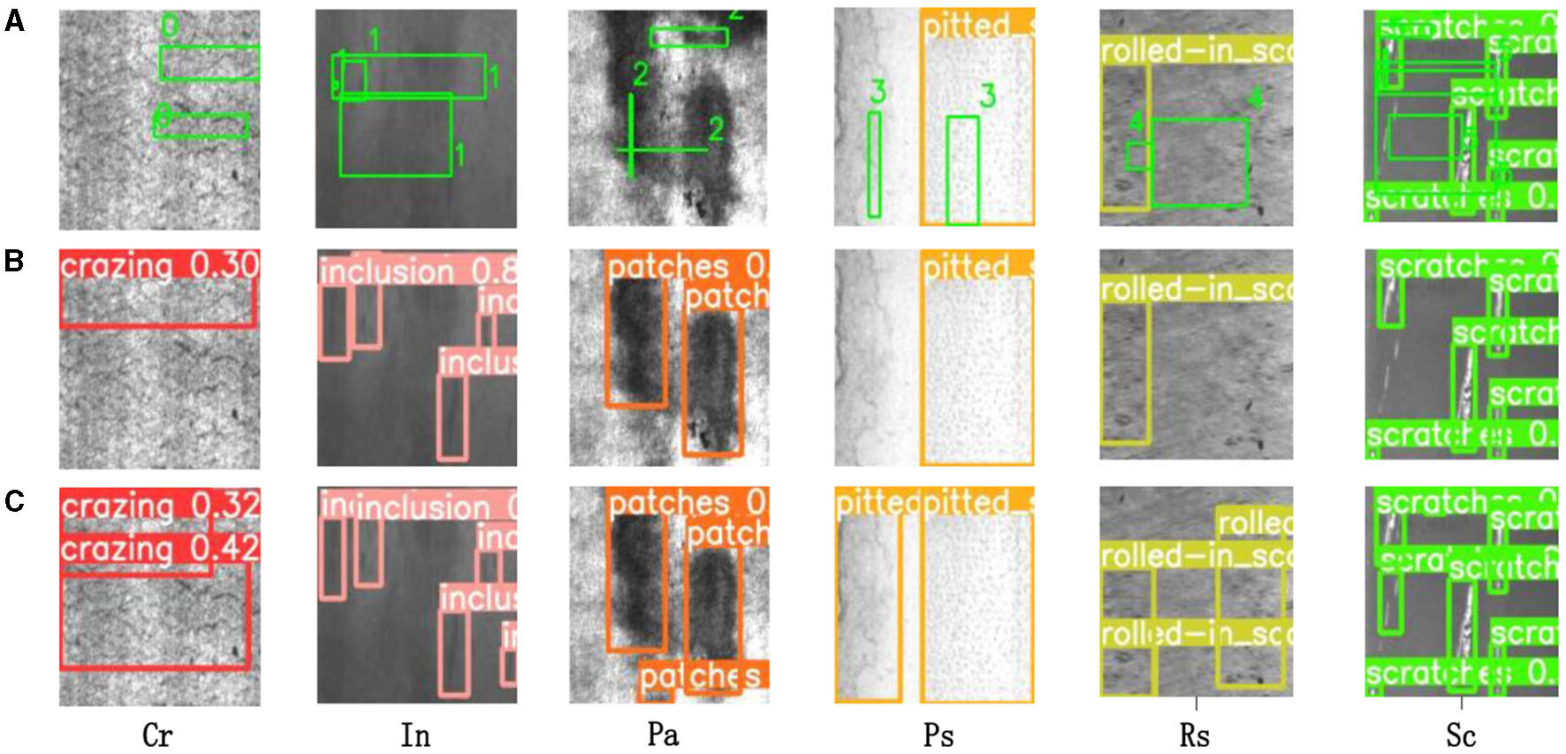
Algorithm detection effect. **(A)** Depicts the original strip steel defect detection image, while **(B, C)** illustrate the detection results of YOLOv5 before and after improvement, respectively. The colored boxes indicate the specific detected defects.

Figure 8A illustrates six classes of original images from the NEU-DET dataset. The images are presented from left to right in the following order: Cr, In, Pa, Ps, Rs, Sc. Figures 8B, C display the corresponding detection results before and after the improvement, respectively. It is evident that YOLOv5-GSS successfully detects all types of defects that were previously undetected by YOLOv5.

#### 4.4.2. Comparison of mainstream attention mechanisms

To validate the effectiveness of the SimAM attention mechanism integrated into the fine neck structure of YOLOv5-GSS, its detection performance is compared with other popular attention mechanisms.

**Figure 9:**
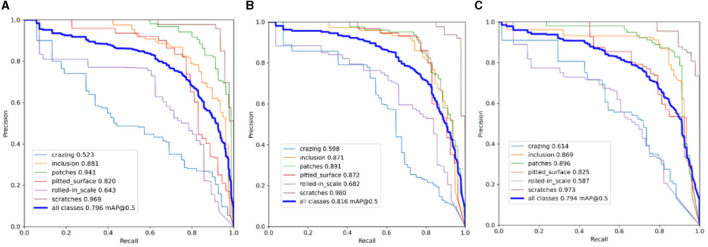
P-R curves for adding attention mechanisms to different structures of the network. **(A)** SimAM in backbone. **(B)** SimAM in neck. **(C)** SimAMin head.

Firstly, it was confirmed that the attention mechanism performed best when added to the neck of the network. Figure 9 displays the detection precision-recall (*P-R*) curves for adding the attention mechanism to the backbone, neck, and detection head, respectively.

The closer the line is to 1 in the P-R plot, the better the detection performance. It is evident from the three plots that the P-R curve in plot (b) converges closer to 1 compared to the other two plots. This suggests that the neck of the network is where the SimAM attention mechanism would work best. SimAM was contrasted with well-known attention mechanisms including SE (Hu et al., 2018), CA (Hou et al., 2021), ECA (Wang et al., 2020), GAM (Liu et al., 2021a), and NAM (Liu et al., 2021b) in order to demonstrate the benefits of including it in the neck of the network. The results in Table 3 demonstrate that, except for the CA (Hou et al., 2021) attention mechanism, all other attention mechanisms exhibit varying degrees of improvement in the network's detection accuracy. Among them, ECA (Wang et al., 2020) shows the most significant improvement, followed by GAMAttention (Liu et al., 2021a) and NAMAttention (Liu et al., 2021b). Comparing these three attention mechanisms, the SimAM attention mechanism demonstrates less pronounced improvement in algorithm accuracy. However, several other attention mechanisms have varying impacts on the model's detection speed, except for the SimAM attention mechanism. Considering the above results, the SimAM attention mechanism stands out due to its combination of a high mAP score, relatively high FPS, and consistent AP% performance across various defect categories. This suggests that SimAM effectively enhances the ability of YOLOv5 to capture and highlight the distinctive features of steel strip surface defects, leading to improved detection accuracy. The choice of attention mechanism often depends on striking a balance between accuracy and efficiency. In this case, SimAM provides a well-rounded improvement in both aspects, making it a favorable option for enhancing defect detection in YOLOv5.

**Table 3:** Comparison of different attention mechanisms.

**Attention**	**mAP%**	**FPS**	**AP%**					
			**Cr**	**In**	**Pa**	**Ps**	**Rs**	**Sc**
SimAM	81.6	105.4	59.6	87.1	89.2	87.4	68.5	98.0
SE (Hu et al., 2018)	81.4	86.2	60.4	88.8	90.3	86	65.9	97.1
CA (Hou et al., 2021)	80.9	58.5	61.3	87.0	92.5	88.9	60.6	95
ECA (Wang et al., 2020)	82.8	68.0	68.8	87.9	92.2	84.9	65.1	97.7
GAM; Liu et al. (2021a)	82.1	49.8	62.4	87.4	91.4	89.6	64.5	97.5
NAM; Liu et al. (2021b)	81.8	70.4	66.9	86.3	91.0	88.4	60.2	97.7
None	80.9	96.2	58.7	85.9	90.7	87.2	65.5	97.7

#### 4.4.3. Comparison with mainstream target detection performance algorithms

To validate the superiority of the YOLOv5-GSS algorithm in terms of detection accuracy and speed, it was compared with other target detection algorithms using the NEU-DET dataset. Firstly, as a lightweight network, YOLOv5-GSS was compared with MobileNetV3 (Howard et al., 2019), ShuffleNetV2 (Ma et al., 2018), YOLOv4-tiny (Bochkovskiy et al., 2020), YOLOX-tiny (Ge et al., 2021), and YOLOv7-tiny (Wang et al., 2022) networks. The results of these comparisons are presented in Tables 4, 5.

**Table 4:** Comparison of the detection speed and accuracy of YOLOv5-GSS with other lightweight networks.

**Model**	**GFLOPs**	**FPS**	**mAP%**	**AP**					
				**Cr**	**In**	**Pa**	**Ps**	**Rs**	**Sc**
Mobilenetv3 (Howard et al., 2019)-YOLOv5	6.40	73.0	80.10	46.3	83.0	95.0	86.3	75.2	94.8
Shuffenetv2 (Ma et al., 2018)-YOLOv5	8.00	90.1	79.30	51.0	87.5	95.0	77.5	70.5	94.5
YOLOv4-tiny (Bochkovskiy et al., 2020)	6.96	145.1	63.60	29.0	80.0	93.0	69.0	44.0	66.0
YOLOX-tiny (Ge et al., 2021)	15.36	60.2	76.96	32.6	81.6	93.0	84.6	76.1	97.3
YOLOv7-tiny (Wang et al., 2022)	13.86	91.9	64.05	16.0	76.0	92.0	80.0	45.0	76.0
YOLOv5-GSS	12.70	100.0	83.80	65.5	86.1	93.0	84.6	76.1	97.3

**Table 5:** Comparison of detection speed and accuracy between YOLOv5-GSS and mainstream target detection algorithm.

**Model**	**GFLOPs**	**FPS**	**mAP%**	**AP%**					
				**Cr**	**In**	**Pa**	**Ps**	**Rs**	**Sc**
(Li et al., 2020)	66.171	50.00	80.10	70.00	88.3	93.7	89.9	76.40	91.70
(Ma et al., 2022)	60.527	91.90	82.40	53.40	77.60	90.20	78.40	71.40	98.10
YOLOv8	28.400	126.6	81.80	64.30	85.90	91.40	84.40	67.20	97.40
YOLOv7	103.20	69.96	73.40	38.10	79.10	92.80	77.30	60.50	92.70
SSD; Kong et al. (2017)	62.747	71.98	74.03	46.60	84.81	94.01	86.46	62.74	69.55
Mask R-CNN (He et al., 2017)	941.17	10.76	81.02	40.96	82.06	93.25	89.97	77.81	93.72
YOLOv5-GSS	12.700	100.0	83.80	65.50	86.10	93.00	84.60	76.10	97.30

Lighter variations of YOLOv4, YOLOv7, and YOLOX are known as YOLOv4-tiny (Bochkovskiy et al., 2020), YOLOv7-tiny (Wang et al., 2022), and YOLOX-tiny (Ge et al., 2021), respectively. MobileNetV3 (Howard et al., 2019) is a lightweight and efficient convolutional neural network architecture, while ShuffleNetV2 (Ma et al., 2018) is a lightweight neural network proposed by the Kuangsi team. The results in Tables 4 demonstrate that YOLOv5-GSS has a clear advantage in detection speed over other lightweight networks and is only slower than YOLOv4-tiny (Bochkovskiy et al., 2020). However, it is important to note that YOLOv4-tiny sacrifices more detection accuracy to achieve this speed advantage. During the experiments, it was observed that the combination of MobileNetV3 (Howard et al., 2019) and YOLOv5 reduced the computational load but also decreased the computation speed. This mismatch can be attributable to the variable computational resource requirements for the detection task across different datasets. The NEU-DET (He et al., 2020) dataset, with its multiple categories and requirement for high detection accuracy, necessitates a greater capacity for feature representation. However, MobileNetV3 (Howard et al., 2019) as the backbone network may not provide sufficient feature representation capacity, resulting in a decrease in computation speed. YOLOv5-GSS appears as the better option for strip steel surface defect identification when the trade-off between detection accuracy and speed is taken into account since it strikes a compromise between the two factors.

A comparison of YOLOv5-GSS with mainstream target detection algorithms is presented in Table 5.

We compared YOLOv5-GSS with Li et al. (2020) and Ma et al. (2022), as well as with YOLOv8, YOLOv7, SSD, and Mask R-CNN (He et al., 2017).

Table 5 provides a comparative evaluation of various object detection models based on several key performance metrics, including computational complexity (GFLOP), inference speed (FPS), mean average precision (mAP), and average precision per class (AP). These metrics offer a clear insight into the characteristics of each model as well as the trade-off between computational efficiency and accuracy.

To verify the significant differences in performance between YOLOv5-GSS and other algorithms, a one-way analysis of variance (ANOVA) was conducted to compare the performance of the algorithms. The *F*-statistic is employed to assess the ratio of between-group variability (SSB) to within-group variability (SSW), where a larger *F*-statistic suggests a greater contribution of between-group variability to the overall variability. The *P*-value serves as an indicator of the statistical significance of the test.

The calculation of between-group variability is given by:


(16) $$\begin{array}{l} SSB=n\overset{k}{\underset{i=1}{\sum }}{\left(\bar{X_{i}}-\bar{X}\right)}^{2} \end{array}$$

where $\bar{X_{i}}$ represents the mean of the i-th group and $\bar{X}$ denotes the overall mean.

The calculation of within-group variability is represented by:


(17) $$\begin{array}{l} SSW=\overset{k}{\underset{i=1}{\sum }}\overset{n}{\underset{j=1}{\sum }}{\left(X_{ij}-\bar{X_{i}}\right)}^{2} \end{array}$$

where*X*_*ij*_ signifies the j-th value of the i-th group and $\bar{X_{i}}$ is the mean of the i-th group.

Consequently, the F-statistic is computed as the ratio of SSB divided by the degrees of freedom between groups (k−1) to SSW divided by the degrees of freedom within groups (nk–k):


(18) $$\begin{array}{l} F=\frac{SSB/\left(k-1\right)}{SSW/\left(nk=k\right)} \end{array}$$

In the context of ANOVA, the null hypothesis assumes equal group means. Through the calculation of the F-statistic, the associated *P*-value is obtained. Specifically, the *P*-value is the minimum of two values:

The tail area of the observed probability distribution of the F-statistic under the assumption of the null hypothesis. This involves looking up critical values or utilizing probability density functions for specific distributions.

The area in the rejection region of the null hypothesis's probability distribution at a given significance level (typically 0.05).

The ANOVA test results in Table 5 yielded an *F*-value of 23.91 and a *P*-value of 0.00000000000444, which is significantly smaller than 0.05. The substantial F-value and exceedingly small *P*-value suggest a statistically significant difference. Consequently, the performance indicators of different models exhibit significant variation. It is evident that there exists a noteworthy distinction between YOLOv5-GSS and other algorithms, underscoring the research significance of the outcomes presented in Table 5.

Improvement models of object detection algorithms in Li et al. (2020) and Ma et al. (2022) demonstrate better detection accuracy and speed. YOLOv8 boasts relatively lower GFLOPs, higher FPS, and a commendable mAP, positioning it as an efficient and accurate choice. In comparison to YOLOv8, YOLOv7 exhibits higher computational complexity. However, this complexity comes at a slight trade-off of reduced FPS and mAP, indicating an imbalance between computational requirements and performance. SSD showcases strong performance in terms of mAP and AP despite its higher computational complexity. Mask R-CNN (He et al., 2017), representing a two-stage object detection algorithm, excels in high detection accuracy, but its significant computational load results in slower detection speed. The table reveals that its detection speed of 10.76 is much lower than that of our algorithm. YOLOv5-GSS stands out with its combination of low GFLOPs, high FPS, and competitive mAP and AP values, highlighting a balanced performance in both accuracy and efficiency.

In conclusion, the distinctiveness of YOLOv5-GSS lies in achieving a strong balance between computational efficiency, real-time inference speed, and accurate object detection. Its low GFLOPs and higher mAP and AP values underscore its suitability for industrial applications such as efficient and accurate detection of surface defects in strip steel, where a combination of efficiency and precision is crucial.

## 5. Conclusion

This paper employs an enhanced lightweight YOLOv5s algorithm for the purpose of detecting surface defects in strip steel. YOLOv5-GSS takes a focused approach by incorporating GSConv and VoV-GSCSP into the feature extraction network and optimizing the neck structure. This targeted enhancement is a unique feature of our approach, ensuring that improvements are applied where they have the most significant impact. The inclusion of the SimAM attention mechanism in the Slim Neck module is a notable innovation. This non-referential and plug-and-play mechanism enhances detection accuracy without introducing complexity, making it a practical addition to the model. Through experimental analysis, it was determined that adding this attention mechanism to the neck of the model yielded the best results. The CIoU loss function was also replaced with SIoU to address issues with the original model. These improvements collectively demonstrate our approach's potential to significantly advance the field of surface defect detection in strip steel while maintaining practicality and efficiency.

To validate the effectiveness of the YOLOv5-GSS algorithm improvements, we conducted a series of ablation experiments and comparative trials. The experimental results demonstrate that the improved model, compared to the original YOLOv5s, achieved a 2.9% increase in mAP and a 3.8 FPS improvement. It outperformed the majority of lightweight networks already in use as well as common target detection methods. The algorithm described in this research is quite helpful in identifying surface flaws in strip steel.

The improved algorithm proposed in this paper has certain limitations that need to be addressed. Specifically, the detection of small targets such as Cr and Rs is still relatively weak. In future research, the focus will be on enhancing the detection of small targets. Additionally, efforts will be made to expand the sample dataset in order to further improve the accuracy of strip surface defect detection while maintaining high detection speed.

## Data availability statement

Publicly available datasets were analyzed in this study. This data can be found here: https://aistudio.baidu.com/aistudio/datasetdetail/195425.

## Author contributions

YZ: Conceptualization, Funding acquisition, Methodology, Project administration, Writing—original draft, Writing—review and editing. SS: Data curation, Formal Analysis, Methodology, Software, Validation, Writing—original draft. SX: Funding acquisition, Project administration, Supervision, Writing—review and editing.
